# In Vitro Assessment of the Combination of Antibiotics against Some Integron-Harbouring Enterobacteriaceae from Environmental Sources

**DOI:** 10.3390/antibiotics11081090

**Published:** 2022-08-11

**Authors:** Folake Temitope Fadare, Elsiddig A. E. Elsheikh, Anthony Ifeanyin Okoh

**Affiliations:** 1 SAMRC Microbial Water Quality Monitoring Centre, University of Fort Hare, Alice 5700, South Africa; 2 Applied and Environmental Microbiology Research Group, Department of Biochemistry and Microbiology, University of Fort Hare, Alice 5700, South Africa; 3 Department of Applied Biology, College of Sciences, University of Sharjah, Sharjah P.O. Box 27272, United Arab Emirates; 4 Department of Environmental Health Sciences, College of Health Sciences, University of Sharjah, Sharjah P.O. Box 27272, United Arab Emirates

**Keywords:** Enterobacteriaceae, in vitro assay, combination therapy, time–kill assay, checkerboard

## Abstract

One strategy for combating antimicrobial resistance in many infections is to combine antibacterial compounds to create combinations that outperform each molecule alone. In this study, we examine and study the inhibitory effect of combining two drugs belonging to different antibiotic classes to obtain a possible potentiating effect against some Enterobacteriaceae isolates harbouring integrons recovered from rivers and effluents of hospital and wastewater treatment plants in Eastern Cape Province, South Africa. These integrons could easily enable the isolates to acquire genes that confer additional resistance against conventional antibiotics. The minimum inhibitory concentration of the various antibiotics was determined using the broth microdilution, while the checkerboard method was used to determine the fractional inhibitory concentration indices (FICIs). A total of 26.3% (10/38) of the interactions were categorised as synergistic, while 73.7% (28/38) were indifferent. None of the combinations were antagonistic. The time–kill assays revealed all the synergistic interactions as bactericidal. Therefore, the combinations of gentamicin with tetracycline, ciprofloxacin, and ceftazidime against multidrug-resistant (MDR) *Klebsiella pneumoniae*, tetracycline–ceftazidime combination against MDR *Escherichia coli*, colistin combinations with ceftazidime and gentamicin, and tetracycline–gentamicin combinations against MDR *Citrobacter freundii* may be future therapeutic alternatives. Hence, the synergistic combinations reported in this study must be assessed further in vivo before their clinical applications.

## 1. Introduction

Antibiotic misuse in clinical and agricultural settings has exacerbated the dissemination of antibiotic-resistant bacteria (ARB) and their resistance determinants in clinical settings and the environment [1,2,3]. Although much research has focused on how antibiotic resistance genes (ARGs) propagate in clinical settings, many papers have also explored how they spread in the environment [2,4,5,6,7]. As a result, various microorganisms, particularly Gram-negative bacteria, acquire resistance against various antibiotics from various antimicrobial classes used to treat the diseases they cause, thus leading to multidrug resistance, which poses a problem for treating future bacterial infections. These ARGs are passed down to offspring or, more typically, through horizontal gene transfer via mobile genetic elements, such as plasmids, bacteriophages, integrons, and transposons. Integrons have a well-established and confirmed involvement in the spread of resistance. They are genetic elements that can capture gene cassettes, which carry various ARGs and serve as expression systems for the genes they harbour [8,9,10].

The emergence of multidrug resistance in Enterobacteriaceae is a critical public health issue that has attracted the attention of the World Health Organization (WHO). They have been classified as one of the critical priority pathogens urgently requiring new antibiotics [11]. The resistance phenomenon has proven most of the current antibiotics ineffective, compounded further by the slow pace of discovery of new antibiotics, necessitating the hunt for new and practical remedies [12,13]. One of such is the exploration of synergy among existing antibiotics. Two medications combined have a higher impact, thereby allowing current antibiotics to be salvaged for use in treating multidrug-resistant (MDR) bacteria, even if the bacteria are resistant against one or both antibiotics separately.

Recognising that no antibiotic compound is universally effective for all illnesses, one of the primary motivations for combining antibiotics was the potential for greater efficacy than single antibiotics. Antibiotics are combined to achieve a variety of goals. The first is the capacity to broaden the antibacterial range during empirical therapy when the pathogen’s identification is still unclear. The second goal is to achieve synergistic effects improving therapeutic efficacy. Other goals include preventing the formation of resistance and reducing host toxicity [14,15,16]. The earliest drugs combined were streptomycin and penicillin in 1950 [17], while trimethoprim and sulphonamides were combined in 1968 [18]. These combinations enhanced the antibiotics’ effectiveness and antibacterial spectrum. Colistin, which, at present, is considered a last-resort drug, functioned well when combined with protein synthesis inhibitors such as linezolid, fusidic acid, and clindamycin, which have minimal effect on Gram-negative bacteria on their own [19]. Now backed by rigorous mechanistic, clinical, and epidemiological data, such combinations remain in frontline use today [16,20,21]. Combinations should be applied against specific life-threatening infections as it has been reported that combinations of antibiotics can also facilitate the spread of resistance. Combining drugs that are not inhibitory but when combined, results in an impact that exceeds the activity of individual drugs owing to complementary activities or various targets of action in microbial cells. Such combinations are effective ways of tackling pathogen-caused diseases. Therefore, our research aims to assess the in vitro activities of various antibiotics from different classes in combination with different antibiotics against environmental strains of integron-harbouring Enterobacteriaceae.

## 2. Results

The minimum inhibitory concentration (MIC) result for each antibiotic assayed against each isolate is presented in Table 1. Sixty-nine per cent (18/26) of the isolates investigated were considered MDR as they exhibited resistance against antibiotics in over two different classes. The highest resistance was observed against ampicillin with a resistance frequency of 73% (19/26), followed by resistance against ceftazidime with 65% (17/26). Others included tetracycline and colistin (58%, 15/26), ciprofloxacin (54%, 14/26), gentamicin (42%, 11/26), and amikacin (15%, 4/26). None of the isolates exhibited resistance against meropenem, considered one of the drugs of last resort, with low MIC values.

All the MDR *Citrobacter* spp. were exposed to various antibiotics for the checkerboard assays, with the combination outcomes shown in Table 2. The outcome of the interactions of the checkboard assays showed that 85% (17/20) of the combinations were indifferent, while 15% (3/20) were synergistic. The synergistic combinations were observed in the combinations of colistin with ceftazidime and gentamicin and between gentamicin and tetracycline. Table 3 shows the outcomes of various antibiotic combinations with interpretable results for *E. coli*, *K. pneumoniae*, and *K. oxytoca*. The most synergistic relationship was observed when gentamicin was combined with tetracycline. The combination of gentamicin with ceftazidime and ciprofloxacin showed a synergistic effect in a *K. pneumoniae* isolate. An *E. coli* isolate also showed synergism with tetracycline and ceftazidime. The FIC index ranged from 0.19 to 1.0. About 9% (7/18) of these exhibited synergistic interactions, while 61% (11/18) were indifferent. No antagonistic reaction was observed in any of the combinations in this study.

**Figure 1:**
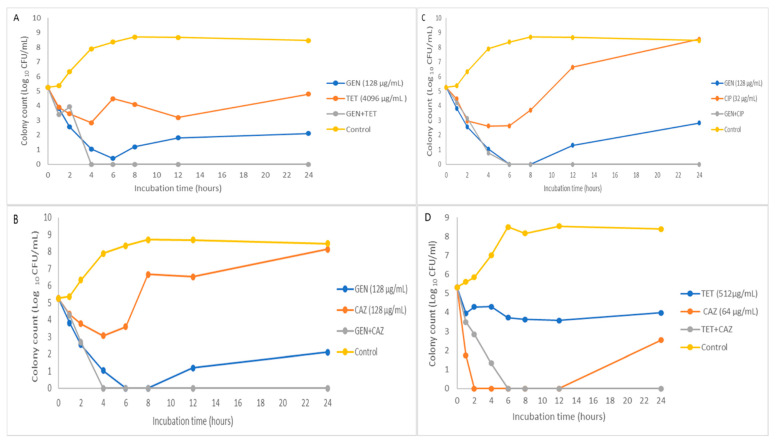
Time-kill curves for antimicrobials in combination at various minimum inhibitory concentration (MIC) values. (**A**–**C**) The combination of gentamicin at MIC with tetracycline, ceftazidime, and ciprofloxacin, respectively, against multidrug-resistant (MDR) *K. pneumoniae* (KP1). (**D**) Drug combination against MDR *E. coli* (E1).

**Figure 2:**
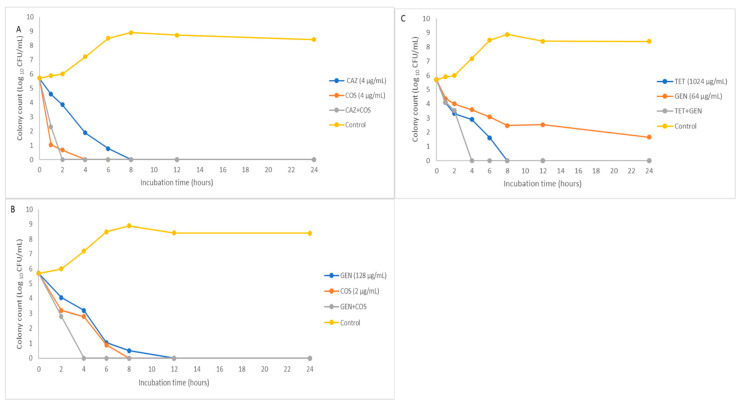
Time–kill curves for antimicrobials in combination at various minimum inhibitory concentration (MIC) values against multidrug-resistant MDR *C. freundii.* (**A**,**B**) The combination of colistin at MIC in combination with ceftazidime and gentamicin against isolates C2 and C4, respectively. (**C**) Drug combination between gentamicin and tetracycline against isolate C4.

The efficacy of these synergistic combinations was further demonstrated in the time–kill curves in Figure 1 and Figure 2. These combinations were bactericidal starting as early as 2 h and maintained throughout the 24 h assay, while synergistic effects were only observed in Figure 1 time–kill curves. In Figure 2, the time–kill curves show the single active agents being bactericidal, although later compared with the combined drugs.

## 3. Discussion

A multidrug-resistant organism displays resistance against a minimum of one antibiotic in more than two different classes [22,23]. Novel approaches to antimicrobial therapy for MDR bacteria have become increasingly crucial as resistance rates to last-resort antibiotics rise. The few antibiotics that are effective against these bacteria have severe clinical limitations, such as hazardous side effects in the case of colistin [24]. Even new agents, such as ceftazidime-avibactam, are susceptible to resistance development [25]. Combination medication regimens are one method of treating MDR Gram-negative bacteria, but little research has been conducted to explore their potency against infections.

All the selected isolates harboured at least one integrase gene, *intI*, and were thus classified as integron-harbouring. Integrons are mobile genetic elements considered efficient gene expression systems that allow bacterial species to capture gene cassettes within their environment and immediately express the ARGs on them due to the presence of inherent promoters [8,26,27]. The presence of integrons with possible ARGs on the gene cassettes further fortifies the bacterial species against the usual antibiotics administered against them. In the previous research conducted by Li and colleagues, integron-harbouring isolates demonstrated resistance against a substantially greater number of antibiotics than negative isolates [28]. Integrons present a selective advantage to bacteria in settings where antibiotic use causes selective pressures, which may explain the high occurrence of multidrug resistance observed in this study.

In this study, meropenem exhibited the lowest MIC values against all the isolates. An outcome that was not unexpected since meropenem is not one of the frontline drugs usually administered against bacterial infections [5,7]. Most of our isolates displayed resistance against more than two different antibiotic classes and were thus classified as multidrug-resistant. These MDR isolates are a concern in the clinical settings and pose a more cause of public health worry when recovered from environmental sources, as in this study. In this era, wherein organisms have acquired various adaptability mechanisms, such as acquiring integrons to survive or evade the arsenal of antibiotics designed against them, other means of combating them must also be devised. Thus, it behoves us to explore the possibilities of combining drugs that can be used simultaneously to combat or reduce the possibility of developing resistance.

In this study, meropenem exhibited the lowest MIC values against all the isolates due to the antibiotic not being one of the frontline drugs usually administered against bacterial infections [5,7]. Most of our isolates displayed resistance against more than two different antibiotic classes and were thus classified as multidrug-resistant. MDR bacteria are usually a concern when recovered in the clinical settings; however, their high detection rate in the environmental settings, as with this study, even poses a greater risk to the public. More commonly now, organisms acquire various adaptability mechanisms, such as the acquisition of integrons to survive or evade the arsenal of antibiotics designed against them, and therefore other means of overcoming this ARB must also be devised. Therefore, it behoves us to explore the possibilities of combining drugs that can be used simultaneously to combat or reduce the possibility of the development of resistance.

The antibiotics with MIC values categorised as resistant were combined in a checkboard style, and the outcomes with interpretable results are shown in Table 2 and Table 3. The various combinations explored yielded synergistic or indifferent interactions of different classes of drugs. As shown in Figure 1A–C, the killing rate of *K. pneumoniae* (KP1) by gentamicin is faster than tetracycline, ciprofloxacin, and ceftazidime. The higher kill rate by gentamicin observed in this study is similar to the results of another in vitro experiment of the ciprofloxacin–gentamicin combination [29]. Our study’s combinations of drugs against MDR *K. pneumoniae* and *E. coli* yielded synergistic and bactericidal outcomes. In Figure 1D, ciprofloxacin’s rate of kill (ROK) was faster than tetracycline’s ROK against *E. coli* (E1). Ciprofloxacin activity was noted to have reduced the cell count to zero as early as 2 h after exposure and was maintained until 12 h, and the viable cells re-emerged at 24 h, suggestive that the drug was a bacteriostatic agent. However, when ciprofloxacin was combined with tetracycline, there were no viable cells from 6 h until the end of the assay, indicative of a bactericidal effect. The synergism observed in the isolates’ time–kill assays (TKAs) further confirms the synergism obtained in the checkerboard assays.

In Figure 2, the ROK of the combined drugs against *C. freundii* isolates are all bactericidal. However, the synergistic interactions obtained in duplicate checkerboard assays were not observed in the TKAs. Except for Figure 2C, where one of the drugs (gentamicin) was not bactericidal throughout the time, most of the single agents were bactericidal at the MIC values. The effectiveness of the combinations was seen at a shorter time to attain the bactericidal effect than the single agents.

The most used antibiotic in the combination studies with interpretable results was gentamicin in this study, as seen in Table 2 and Table 3. It belongs to the aminoglycoside class of antibiotics and is used in treating MDR bacteria. Although they have been used for several decades to treat infections caused by non-fastidious Gram-negative bacteria [30], the most prevalent bacterial resistance mechanisms in this antibiotic class are the enzymatic modification aminoglycoside antibiotics [31]. The enzymes belong to families, such as aminoglycoside phosphotransferases (APHs), aminoglycoside acetyltransferases (AACs), and aminoglycoside adenyl transferases (ANTs) [32,33]. These enzymes are often encoded on gene cassettes of integrons, also present in the isolates investigated in this study.

In this study, one of the combinations of gentamicin with ceftazidime yielded a synergistic interaction, and further investigation in the TKA revealed the bactericidal and synergistic effects of the combination compared to the individual agents. Here, *K. pneumoniae* (KP1) was resistant against ceftazidime, while gentamicin showed better activity but was not bactericidal against the isolate. It has been reported that β-lactams, such as ceftazidime, are known to break the bacterial cell wall in a non-fatal way, allowing aminoglycosides, such as gentamicin, to enter bacteria and increase their killing effectiveness [33,34]. In another study, aminoglycosides, due to their synergistic antibacterial properties, were combined with β-lactam antibiotics, which broadened the scope of treatment, accelerated bacterial clearance and enhanced antibiotic resistance [35]. Several other studies have reported the combination of aminoglycosides with β-lactams for treating MDR bacteria species [33,36,37].

In this study, the combination of gentamicin (aminoglycoside) with colistin (polymyxins) against *C. freundii*, which yielded a synergistic interaction, as shown in Table 2, is similar to the reports of Hussein and colleagues, where amikacin was combined with polymyxins with a synergistic antibacterial effect [38].

In vitro assessments of antimicrobial synergy are naturally limited in their ability to predict in vivo outcomes accurately; hence, necessary precautions must be taken to apply such combination therapy in clinical applications. First, the concentrations tested may be above the tolerable threshold for the actual serum levels, and a pharmacokinetic/pharmacodynamic simulation is needed. As far as in vivo study is concerned, higher MIC levels for the antibiotics tested may not be clinically beneficial. Another critical concern is that the inoculum size used for these in vitro assays may differ significantly in vivo vis-a-viz host defence mechanisms, and the checkerboard results and rate of kill assays obtained in this study may not reflect the accurate outcome when utilised in clinical settings. Therefore, future studies in which the synergistic and bactericidal relationships observed in the combinations in this present study need to be tested in animal models, pharmacokinetic/pharmacodynamic studies, and human subjects will be essential in determining the possible clinical outcomes applications of our findings. Although, within the confines of in vitro studies, specific steps were taken to increase the robustness of our results by testing different isolates in the checkerboard array and then further assessing the synergistic combinations through the TKAs. In most cases, synergy was also present in the ROK studies, and all the synergistic relationships from the checkboard assays were bactericidal (Figure 1 and Figure 2).

## 4. Materials and Methods

### 4.1. Bacterial Isolate Characterisations

Enterobacteriaceae isolates were selected from our previous studies [4,5,22], with the various sources indicating the diversity of the environmental isolates assessed (Table 1). These isolates were deposited in the Applied and Environmental Microbiology Research Group (AEMREG) culture collection. The bacterial strains were resuscitated in Brain Heart Infusion (BHI) broth (Merck, Johannesburg, South Africa) and incubated at 37 ± 1 °C for 18 ± 2 h. A loopful was streaked on Violet Red Bile Glucose (VRBG) agar incubated overnight at 37 ± 1 °C. Isolates were purified further by streaking twice on nutrient agar (Oxoid, Basingstoke, UK). Single pure colonies were transferred to 2 mL BHI broth and genomic DNA was extracted using the boiling method previously described [39]. The identities of the isolates were confirmed using conventional polymerase chain reaction (PCR). The integrase genes (*intI1* and *intI2*) were assayed to classify the integrons present in the confirmed isolates. The list of primers and thermocycling conditions for the PCR assays are presented in Supplementary Table S1.

### 4.2. Preparation of Antibiotics and Media Used

Standard laboratory powders assayed included ceftazidime, gentamicin, tetracycline, ciprofloxacin, colistin sulphate, ampicillin, meropenem, and amikacin. These were purchased from Sigma-Aldrich (St. Louis, MO, USA). The stock solutions were prepared using the potency (µg per mg powder) of each antibiotic as supplied by the manufacturer, following the formula below [13,40]:

$$W=\frac{C\times V}{P}$$

where ‘*W*’ is the weight of the antibiotics to be dissolved (mg), *C* is the desired concentration of the stock solution to be prepared (µg/mL), *V* is the desired volume (mL), and *P* is the potency of the antibiotic powder as supplied by the manufacturer (µg/mg). The diluent of all antibiotics used was sterilised distilled water, while anhydrous sodium carbonate at 10% weight was added to ampicillin and ceftazidime stock solutions [41]. We used double-strength Muller Hinton II Broth (2× MHB) (Oxoid, Basingstoke, UK).

### 4.3. Standardisation of Inoculum

Following the guidelines recommended by the Clinical and Laboratory Standard Institute (CLSI), the inoculum was prepared by adjusting the turbidity of the test microorganisms in sterilised normal saline to 0.5 McFarland using the spectrophotometer (Merck), with a wavelength set at 600 nm. The absorbance of the test microorganisms ranged from an optical density of 0.08–0.1 to produce an approximate 1 × 10^8^ CFU/mL inoculum size. Then, 0.1 mL of the adjusted 0.5 McFarland standard inoculum was transferred to 9.9 mL 2× MHB to give an approximate 1 × 10^6^ CFU/mL inoculum size used within 30 min to avoid a change in cell number [41].

### 4.4. Antimicrobial Susceptibility Testing

The MIC of the antibiotics was determined using the round-bottomed 96-well microtiter plates (Greiner Bio-one, Monroe, NC, USA) following the broth microdilution procedure described by Wiegand and colleagues [40]. Briefly, 50 µL of sterile distilled water was aliquoted into the wells 2 to 10, which served as the antibiotics’ diluent. Subsequently, 100 µL of the highest concentration of the antibiotics to be investigated was dispensed into well 1. It was serially diluted by transferring 50 µL of the antibiotics from well 1 through well 10 and finally discarded after dilution in the last well allowing for the geometric serial dilution of the antibiotics across the rows. Each well containing the antibiotic solution was inoculated with 50 µL of the test organism earlier standardised. Well 11 served as the growth control (GC), containing only the inoculum, while well 12 served as the sterility control (SC), only containing the assayed antibiotics.

The microtiter plates were covered and incubated at 35 ± 1 °C for 16–20 ± 2 h. The results were read after the addition of the 30 µL resazurin dye (*w*/*v*, 0.015%) (Glentham Life Sciences, Corsham, UK) or the 2,3,5, triphenyl tetrazolium chloride (Merck, Darmstadt, Germany), depending on the availability of the dyes, with a further 2 h incubation period for the observation of a colour change. The well with the lowest concentration of the antibiotics that completely inhibited the growth of the bacteria, as indicated by no observable colour change, was read as the MIC value, which was interpreted according to the European Committee on Antimicrobial Susceptibility Testing (EUCAST) and CLSI breakpoints [41,42]. The tests were performed in triplicates.

### 4.5. Quality Control

Quality control was performed to validate the methods employed in this study. The performance of all the antibiotic stock solutions was validated against referenced organisms *Escherichia coli* ATCC 25922, *Staphylococcus aureus* ATCC 25923, and *Pseudomonas aeruginosa* ATCC 27853. The reference strains were purchased from the American Type Culture Collection (Manassas, VA, USA) to determine the MIC values. The results were compared with EUCAST values [42].

Immediately after the inoculation of the microtiter plates with the inoculum for the MIC studies, 10 µL of the bacterial inoculum were obtained from the GC columns (columns without antibiotics) and aliquot into sterile 990 µL 2× MHB vortexed to ensure they were thoroughly mixed; from this, another 1:100 dilution was produced. Then, 100 µL from each dilution were aseptically spread onto sterile Muller Hinton agar (MHA) plates and incubated at 35 ± 1 °C. Colonies were counted after 18 ± 2 h, and values obtained from around 50 colonies on the lower dilution indicated that bacterial inoculum was accurately standardised [40].

### 4.6. Checkerboard Assay

Antibiotics from different classes whose breakpoints were non-susceptible were combined in a checkerboard style for this assay. First, each antibiotic was prepared by serially diluting in water to obtain the desired dilution folds starting from double the MIC values obtained earlier. A total of 50 µL of drug A was dispensed down each column starting from the highest concentration except for column 12. Similarly, drug B was dispensed along the rows except for row H. Then, 50 µL of each adjusted 0.5 McFarland standard was transferred into 15 mL 2× MHB and aliquoted to all the wells to obtain a final concentration of 5 × 10^5^ CFU/mL with a final volume of 150 µL per well. The last well, H12, served as the GC. The results were obtained after 24 ± 2 h of incubation at 35 ± 1 °C as described earlier. The fractional inhibitory concentration (FIC) index of the combined drugs was calculated as follows:

$$\frac{\mathrm{MIC}\;A\left(\mathrm{combination}\right)}{\mathrm{MIC}\;A\left(\mathrm{alone}\right)}+\frac{\mathrm{MIC}\;B\left(\mathrm{combination}\right)}{\mathrm{MIC}\;B\left(\mathrm{alone}\right)}=\mathrm{FIC}\;A+\mathrm{FIC}\;B=\mathrm{FIC}\;\mathrm{Index}$$

Synergy was defined as an FIC index value less than 0.5, while antagonism was defined for values greater than 4, and values in between were interpreted as indifferent [19,43]. The assays were duplicated, and synergy was determined when the FICI yielded values less than 0.5. When a skipped well occurred, the higher FICI was used to prevent false-positive synergy interpretations. The data were discarded if there were more than two skipped wells in a single grid or if the MIC was more than a 2-fold dilution above or below the modal MIC for that isolate, followed by a repeat of the experiment. However, the antibiotic combination for the isolate was eliminated from further investigation if the same error persisted [19].

### 4.7. Time-Kill Assays 

The time-kill assays (TKAs) were performed on all the synergistic combinations from the checkboard assays. For different isolates that demonstrated synergy to the same antibiotic combinations, only one was selected for the TKA. The kill rate was determined by enumerating the viable cell counts at specific intervals over 24 ± 2 h. The MICs of each antibiotic alone and the combined antibiotics at 20 mL each were investigated in a 100 mL conical flask. Then, 200 µL of the adjusted 0.5 McFarland inoculum was added to 20 mL of 2× MHB to produce a final concentration of 5 × 10^5^ CFU/mL when added to the antibiotics to be assayed. The cultures were incubated at 35 ± 1 °C with shaking at 120 rpm. Aliquots were removed from the cultures at 0, 1, 2, 4, 6, 8, 12, and 24 h and a 10-fold dilution series was performed in sterile 2× MHB. A 100 µL of each appropriate dilution was spread on MHA plates in triplicates. The plates were incubated at 35 ± 1 °C, and colony counts were recorded after 24 ± 2 h. A growth control was run in parallel with each experiment. The time–kill curves were determined by plotting the mean colony counts (log CFU/mL) against the incubation time (hours). The combination’s efficacy was synergistic when viable bacteria were reduced by ≥2 log10 CFU/mL compared to the most active single antibiotic. The combination therapy’s efficiency was also evaluated as bactericidal when there was a ≥3 log10 CFU/mL reduction compared to the initial inoculum at 24 ± 2 h.

### 4.8. Data Analysis

Data were entered on Microsoft Excel 2016 and statistical analysis was performed using descriptive analysis.

## 5. Conclusions

According to the findings of this study, some in vitro combinations of different classes of antibiotics targeting different mechanisms of action can be effective against MDR Enterobacteriaceae infections. However, further studies, including pharmacokinetics, pharmacodynamics, and clinical trials, are needed on the synergistic combinations to confirm their advantages over monotherapy.

## Figures and Tables

**Table 1:** Bacterial species characterisation, including minimum inhibitory concentration (MIC).

Isolate Identifier	Species	Source ^b^	Integrase Gene			MIC ^a^ (µg/mL)					
				GEN	MEM	CIP	TET	CAZ	AMP	COS	AMK
C1	*C. braakii*	WWTP	*intI1* + *intI2*	1	0.03	1	1024	64	>4096	4	1
C2	*C. freundii*	River	*intI1*	2	0.015	0.25	1	2	16	4	4
C3	*C. freundii*	HWW	*intI1*	64	0.03	64	128	128	>4096	4	4
C4	*C. freundii*	HWW	*intI1*	128	0.015	128	1024	128	>4096	2	16
EC1	*E. cloacae*	River	*intI1*	1	0.015	0.06	4	0.05	4	0.125	2
EC2	*E. cloacae*	WWTP	*intI1*	1	0.015	0.06	2	0.05	4	0.25	2
EC3	*E. cloacae*	WWTP	*intI1* + *intI2*	2	0.25	2	64	8	≤8	4	8
E1	*E. coli*	River	*intI1* + *intI2*	32	0.015	0.03	512	32	>4096	8	8
E2	*E. coli*	WWTP	*intI1* + *intI2*	128	0.007	1	1024	128	>4096	4	8
E3	*E. coli*	HWW	*intI1* + *intI2*	64	0.007	2	512	64	1024	8	64
E4	*E. coli*	WWTP	*intI1* + *intI2*	1	0.007	0.06	4	0.5	8	0.125	4
KO1	*K. oxytoca*	WWTP	*intI1*	1	0.125	>32	8	8	>4096	1	1
KO2	*K. oxytoca*	HWW	*intI1*	1	0.015	≤0.06	4	≤0.5	16	1	4
KO3	*K. oxytoca*	HWW	*intI1*	128	0.06	16	64	˃256	>4096	64	32
KO4	*K. oxytoca*	HWW	*intI1*	8	0.125	64	512	64	8192	0.5	0.5
KO5	*K. oxytoca*	HWW	*intI1*	0.5	0.03	≤0.003	2	0.25	8	1	0.25
KO6	*K. oxytoca*	WWTP	*intI1* + *intI2*	0.25	0.015	≤0.0019	1	0.06	64	2	0.125
KP1	*K. pneumoniae*	WWTP	*intI1*	128	0.125	32	4096	128	>4096	8	8
KP2	*K. pneumoniae*	WWTP	*intI1*	0.5	0.015	0.03	4	0.5	8	4	0.5
KP3	*K. pneumoniae*	River	*intI1* + *intI2*	1	0.015	0.5	2	32	1024	4	4
KP4	*K. pneumoniae*	River	*intI1* + *intI2*	1	0.015	0.125	2	1	32	1	2
KP5	*K. pneumoniae*	WWTP	*intI1*	32	0.25	16	1024	512	>16384	>4096	1
KP6	*K. pneumoniae*	HWW	*intI1*	16	0.03	64	512	128	8192	0.5	8
KP7	*K. pneumoniae*	HWW	*intI1*	1	0.06	0.015	54	0.5	8	>4096	32
KP8	*K. pneumoniae*	HWW	*intI1*	1	0.015	0.125	1	64	>4096	8	2
KP9	*K. pneumoniae*	HWW	*intI1* + *intI2*	128	0.03	2	128	64	>4096	4	2

^a^ The shaded portions indicate MIC values classified as resistant, while the unshaded areas indicate those classified as susceptible. GEN: gentamicin, MEM: meropenem, CIP: ciprofloxacin, TET: tetracycline, CAZ: ceftazidime, AMP: ampicillin, COS: colistin, and AMK: amikacin. ^b^ Source of bacterial isolation includes WWTP: wastewater treatment plant effluents, HWW: hospital wastewater effluents.

**Table 2:** The minimum inhibitory concentration (MIC) of various antibiotics alone and the results of the checkerboard assays for multidrug-resistant integron-harbouring *Citrobacter* spp.

Isolate Identifier	Antibiotic ^a^	MIC Alone	MIC in Combination	FIC ^b^	FICI ^c^	Interpretation
C1	CAZ	128	64	0.50	1.00	Indifferent
	COS	2	1	0.50		
C2	CAZ	4	1	0.25	0.50	Synergy
	COS	4	1	0.25		
C3	CAZ	128	64	0.50	1.00	Indifferent
	COS	2	1	0.50		
C4	CAZ	128	32	0.25	0.75	Indifferent
	COS	2	1	0.50		
C1	TET	1024	512	0.50	0.75	Indifferent
	GEN	1	0.25	0.25		
C2	TET	2	1	0.50	1.00	Indifferent
	GEN	2	1	0.50		
C3	TET	256	16	0.06	0.56	Indifferent
	GEN	32	16	0.50		
C4	TET	1024	128	0.13	0.25	Synergy
	GEN	64	8	0.13		
C1	GEN	1	0.5	0.50	0.75	Indifferent
	COS	2	0.5	0.25		
C2	GEN	1	0.25	0.25	0.75	Indifferent
	COS	4	2	0.50		
C3	GEN	64	32	0.50	0.63	Indifferent
	COS	2	0.25	0.13		
C4	GEN	128	32	0.25	0.38	Synergy
	COS	2	0.25	0.13		
C1	TET	2048	1024	0.50	0.75	Indifferent
	COS	2	0.5	0.25		
C2	COS	4	4	1.00	2.00	Indifferent
	TET	2	2	1.00		
C3	TET	128	64	0.50	1.00	Indifferent
	COS	2	1	0.50		
C4	TET	2048	1024	0.50	0.75	Indifferent
	COS	2	0.5	0.25		
C1	AMP	8192	4096	0.50	1.00	Indifferent
	CIP	0.5	0.25	0.50		
C2	AMP	8	4	0.50	1.00	Indifferent
	CIP	0.25	0.125	0.50		
C3	AMP	8192	4096	0.50	1.00	Indifferent
	CIP	2	1	0.50		
C4	AMP	8192	4096	0.50	1.00	Indifferent
	CIP	128	64	0.50		

^a^ Antibiotic codes: GEN: gentamicin, MEM: meropenem, CIP: ciprofloxacin, TET: tetracycline, CAZ: ceftazidime, AMP: ampicillin, COS: colistin, and AMK: amikacin. ^b^ FIC represents the fractional inhibitory concentration of each drug calculated as MIC in combination/MIC alone. ^c^ FICI represents the fractional inhibitory concentration index of both drugs calculated by adding the FIC of the two drugs.

**Table 3:** The minimum inhibitory concentration (MIC) of antibiotics singly and in combination as derived from the checkerboard assays for multidrug-resistant integron-harbouring *E. coli*, *K. pneumoniae,* and *K. oxytoca*.

Organism (Isolate Code)	Antibiotic ^a^	MIC Alone	MIC in Combination	FIC ^b^	FICI ^c^	Interpretation
*E. coli* (E3)	GEN	32	8	0.25	0.50	Synergy
	TET	512	128	0.25		
*E. coli (E2*)	GEN	64	16	0.25	0.38	Synergy
	TET	512	64	0.13		
*E. coli* (E1)	GEN	64	16	0.25	0.31	Synergy
	TET	1024	64	0.06		
*K. pneumoniae* (KP1)	GEN	128	16	0.13	0.38	Synergy
	TET	4096	1024	0.25		
*K. oxytoca* (KO1)	GEN	0.5	0.06	0.12	0.62	Indifferent
	TET	4	2	0.50		
*E. coli* (E3)	GEN	32	16	0.50	0.63	Indifferent
	CAZ	128	16	0.13		
*E. coli (E2*)	GEN	64	16	0.25	0.75	Indifferent
	CAZ	64	32	0.50		
*E. coli* (E1)	GEN	64	32	0.50	0.75	Indifferent
	CAZ	64	16	0.25		
*K. pneumoniae* (KP1)	GEN	128	16	0.13	0.19	Synergy
	CAZ	128	8	0.06		
*K. pneumoniae* (KP1)	GEN	128	16	0.13	0.38	Synergy
	CIP	32	8	0.25		
*K. pneumoniae* (KP1)	CAZ	128	64	0.50	1.00	Indifferent
	CIP	32	16	0.50		
*K. pneumoniae* (KP1)	AMP	16384	8192	0.50	1.00	Indifferent
	CIP	32	16	0.50		
*E. coli* (E3)	TET	512	256	0.50	0.63	Indifferent
	CAZ	128	16	0.13		
*E. coli (E2*)	TET	512	256	0.50	1.00	Indifferent
	CAZ	64	32	0.50		
*E. coli* (E1)	TET	512	64	0.13	0.38	Synergy
	CAZ	64	16	0.25		
*K. oxytoca* (KO1)	TET	4	1	0.25	0.75	Indifferent
	COS	0.5	0.25	0.50		
*K. oxytoca* (KO1)	CAZ	8	2	0.25	0.75	Indifferent
	COS	0.5	0.25	0.50		
*K. oxytoca* (KO1)	CIP	256	128	0.50	0.75	Indifferent
	COS	0.5	0.125	0.25		

^a^ Antibiotic codes: GEN: gentamicin, MEM: meropenem, CIP: ciprofloxacin, TET: tetracycline, CAZ: ceftazidime, AMP: ampicillin, COS: colistin, and AMK: amikacin. ^b^ FIC represents the fractional inhibitory concentration of each drug calculated as MIC in combination/MIC alone. ^c^ FICI represents the fractional inhibitory concentration index of both drugs calculated by adding the FIC of the two drugs.

## Data Availability

Not applicable.

## Supplementary Materials

The following supporting information can be downloaded at: https://www.mdpi.com/article/10.3390/antibiotics11081090/s1, Table S1: Primer sequences and thermocycling conditions used in this study. References [44,45,46,47,48,49,50,51] are cited in the supplementary materials.
