# Supplementary material for: In Vitro Assessment of the Combination of Antibiotics against Some Integron-Harbouring Enterobacteriaceae from Environmental Sources
Source: Antibiotics (Basel). 2022 Aug 11;11(8):1090. doi: 10.3390/antibiotics11081090 (PMC9404769; doi:10.3390/antibiotics11081090)
Supplement: Supplementary file 1 [file antibiotics-11-01090-s001.zip › antibiotics-1841720-supplementary.pdf]

Supplementary Table S1: Primer sequences and thermocycling conditions used in this study.

Target strain	Target gene	Primer Sequence (5′ →3′)	Amplicon size (bp)	Thermocycling conditions	Reference
Klebsiella genus	*gryA*	F: CGCGTACTATACGCCATGAAGTA<br>R: ACCGTTGATCACTTCGGTCAGG	441	94 °C for 5 mins; 35 [94 °C, 30 s; 55 °C, 45 s; 72 °C, 45s]; 72 °C for 7 min.	[1]
Klebsiella pneumoniae	*16S-23S ITS*	F: ATT TGA AGA GGT TGC AAA CGA T<br>R: TTC ACT CTG AAG TTT TCT TGT GTT C	130	94 °C for 5 mins; 30 [94 °C, 30 s; 55 °C, 30 s; 72 °C, 40 s]; 72 °C for 10 min.	[2]
Klebsiella oxytoca	*pehX*	F: GATACGGAGTATGCCTTTAC-GGTG<br>R: TAGCCTTTATCAAGCGGA-TACTGG	343	94 °C for 5 mins; 30[94 °C, 30 s; 55 °C, 30 s; 72 °C, 40 s]; 72 °C for 10 min.	[3]
Enterobacter cloacae	*hsp60*	F: GTAGAAGAAGGCGTGGTTGC R: ATGCATTCGGTGGTGATCATCAG	341	94 °C for 5 mins; 30 [94 °C, 30 s; 54 °C, 30 s; 72 °C, 1 min]; 72 °C for 5 min.	[4]
Citrobacter genus	*Citrobacter urease*	F: TGAAGCTGAACTACCCGGAATC R: TGTCCAGGCTCAAAACGTAC	454	94 °C for 4 mins; 30 [94 °C, 30 s; 55 °C, 40 s; 72 °C, 1 min]; 72 °C for 7 min.	[5]
Escherichia coli	uidA	F: AAAACGGCAAGAAAAAGCAG R: ACGCGTGGTTACAGTCTTGCG	147	94 °C for 5 mins; 30[94 °C, 30 s; 58 °C, 1 min; 72 °C, 1 min]; 72 °C for 8 min.	[6]
Class 1 in-tegron	*intI1*	F: CAGTGGACATAAGCCTGTTC<br>R: CCCGAGGCATAGACTGTA	164	94 °C for 5 mins; 35[94 °C, 60 s; 55 °C, 60 s, 72 °C, 30 s] 72 °C, 10 min	[7]
Class 2 in-tegron	*intI2*	F: TTATTGCTGGGATTAGGC<br>R: ACGGCTACCCTCTGTTATC	232	94 °C for 5 mins; 32[94 °C, 60 s; 59 °C, 60 s; 72 °C, 2 mins]; 72 °C for10 mins.	[8]

References

1. Brisse, S.; Verhoef, J. Phylogenetic diversity of Klebsiella pneumoniae and Klebsiella oxytoca clinical isolates revealed by randomly amplified polymorphic DNA, gyrA and parC genes sequencing and automated ribotyping.. *Int. J. Syst. Evol. Microbiol.* **2001**, *51*, 915–924, https://doi.org/10.1099/00207713-51-3-915.
2. Liu, Y.; Liu, C.; Zheng, W.; Zhang, X.; Yu, J.; Gao, Q.; Hou, Y.; Huang, X. PCR detection of Klebsiella pneumoniae in infant formula based on 16S–23S internal transcribed spacer. *Int. J. Food Microbiol.* **2008**, *125*, 230–235, https://doi.org/10.1016/j.ijfoodmicro.2008.03.005.
3. Kovtunovych, G.; Lytvynenko, T.; Negrutska, V.; Lar, O.; Brisse, S.; Kozyrovska, N. Identification of Klebsiella oxytoca using a specific PCR assay targeting the polygalacturonase pehX gene. *Res. Microbiol.* **2003**, *154*, 587–592, https://doi.org/10.1016/s0923-2508(03)00148-7.
4. Akbari, M.; Bakhshi, B.; Peerayeh, S.N. Particular Distribution of Enterobacter cloacae Strains Isolated from Urinary Tract Infection within Clonal Complexes. *Iran. Biomed. J.* **2015**, *20*, 49–55, https://doi.org/10.7508/ibj.2016.01.007.
5. Lü, A.; Hu, X.; Zheng, L.; Zhu, A.; Cao, C.; Jiang, J. Isolation and characterization of Citrobacter spp. from the intestine of grass carp Ctenopharyngodon idellus. *Aquaculture* **2011**, *313*, 156–160, https://doi.org/10.1016/j.aquaculture.2011.01.018.
6. Bej, A.K.; DiCesare, J.L.; Haff, L.; Atlas, R.M. Detection of Escherichia coli and Shigella spp. in water by using the polymerase chain reaction and gene probes for uid. *Appl. Environ. Microbiol.* **1991**, *57*, 1013–1017, https://doi.org/10.1128/aem.57.4.1013-1017.1991.
7. Koeleman, J.G.M.; Stoof, J.; Van Der Bijl, M.W.; Vandenbroucke-Grauls, C.M.J.E.; Savelkoul, P.H.M. Identification of Epidemic Strains of *Acinetobacter baumannii* by Integrase Gene PCR. *J. Clin. Microbiol.* **2001**, *39*, 8–13, https://doi.org/10.1128/jcm.39.1.8-13.2001.
8. Goldstein, C.; Lee, M.D.; Sanchez, S.; Hudson, C.; Phillips, B.; Register, B.; Grady, M.; Liebert, C.; Summers, A.; White, D.G.; et al. Incidence of Class 1 and 2 Integrases in Clinical and Commensal Bacteria from Livestock, Companion Animals, and Exotics. *Antimicrob. Agents Chemother.* **2001**, *45*, 723–726, https://doi.org/10.1128/aac.45.3.723-726.2001.
